# Interactions of Aminopropyl–Azithromycin Derivatives, Precursors in the Synthesis of Bioactive Macrozones, with *E. coli* Ribosome: NMR and Docking Studies

**DOI:** 10.3390/ma14195561

**Published:** 2021-09-25

**Authors:** Ivana Mikulandra, Tomislav Jednačak, Branimir Bertoša, Jelena Parlov Vuković, Iva Kušec, Predrag Novak

**Affiliations:** 1 Department of Chemistry, Faculty of Science, University of Zagreb, Horvatovac 102a, HR-10000 Zagreb, Croatia; ivana.mikulandra@chem.pmf.hr (I.M.); tjednacak@chem.pmf.hr (T.J.); branimir.bertosa@chem.pmf.hr (B.B.); kuseciva38@gmail.com (I.K.); 2 INA-Industrija Nafte d.d., Refining & Marketing, Central Testing Laboratory, Lovinčićeva bb, HR-10002 Zagreb, Croatia; Jelena.Parlov-Vukovic@ina.hr

**Keywords:** azithromycin derivatives, interactions, ribosome-bound epitopes, NMR spectroscopy, docking

## Abstract

The structure and interactions of several aminopropyl–azithromycin derivatives (**1a**–**c**) have been studied by using NMR spectroscopy and docking calculations. Compounds **1a**–**c** are precursors in the synthesis of macrozones, novel bioactive azithromycin–thiosemicarbazone conjugates active against some resistant bacterial strains. Today, bacterial resistance is considered as one of the major threats to human health. Knowledge on drug binding mode and conformations is one of the key factors in the process of designing molecules to fight resistance. In solution state, compounds **1a** and **1c** exist in the 3-*endo*-folded-out conformation, while **1b** adopts a classical folded-out conformation. ^13^C and ^15^N CPMAS NMR spectra pointed towards similar structures in the solid state. The transferred NOESY NMR spectra confirmed binding to the *E. coli* ribosome and suggest that dominant conformations in the bound state resemble those in the free one. STD experiments identified reactive groups of **1a**–**c** in close contact with the ribosome resembling binding epitopes observed for the related 15-membered macrolides. Docking studies revealed that the studied compounds bind to the same ribosome binding pocket similarly to erythromycin in the crystal state, and that the binding is achieved through H-bonds and van der Waals interactions. The bound conformation is the same as determined by NMR. STD enhancements observed for methylene protons in the aminopropyl side chain indicate additional interactions which contribute to the overall binding energy.

## 1. Introduction

Macrozones belong to a novel class of azithromycin–thiosemicarbazone conjugates that exhibit very good antibacterial activities against both susceptible and some resistant bacterial strains, such as efflux- and MLS (macrolides–lincosamides–streptogramines)- resistant *S. aureus*, *S. pneumoniae* and *S. pyogenes* strains [1,2]. Macrolide antibiotics, such as azithromycin, have been in clinical use for over 50 years, mostly due to their high efficacy, safety and favorable pharmacokinetics. They are prescribed to treat upper and lower respiratory tract infections [3]. Macrolides bind to the 23S rRNA of the 50S subunit, near or at the peptidyl transferase center (PTC), block the nascent peptides exit tunnel and thus inhibit the synthesis of bacterial proteins. However, emerging bacterial resistance to antibiotics compromises their use as effective antimicrobials and represents a serious threat to human health worldwide. There are several resistance mechanisms to macrolides and the most frequent are modifications in the ribosome target, efflux pumps, inactivation of the compound and mutations in the 23S rRNA and some proteins [4]. Therefore, the discovery of novel and safer macrolides active against resistant pathogenic bacteria is today an ultimate goal of antibiotic drug design.

**Scheme 1:**
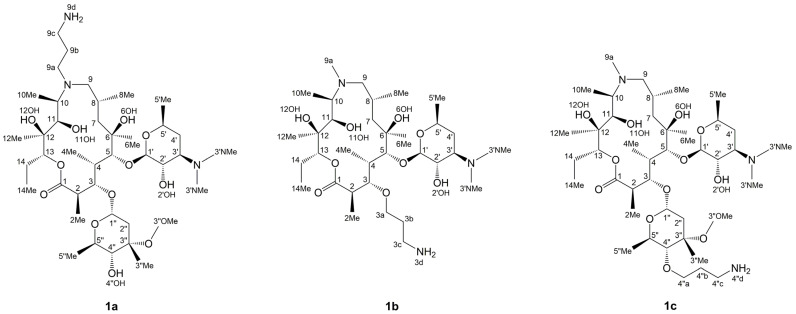
Structure and atom numbering of aminopropyl–azithromycin derivatives, precursors in the synthesis of macrozones.

As above mentioned, macrozones are novel hybrid azithromycin derivatives designed to overcome bacterial resistance [1,2]. Structurally, they consist of a polyfunctional macrolactone ring with two saccharide units (desozamine and cladinose) and a thiosemicarbazone moiety attached to it at certain positions (Scheme 1).

A crucial step in the discovery of novel compounds for preventing resistance is to understand how macrolides interact with their biological target ribosome. Crystal structures of some ribosome–macrolide complexes have been solved [5,6,7] and they elucidate important binding mechanisms of macrolides to ribosomes. However, we believe that the process of new antibiotic design should also include elucidation of the solution-state structures and dynamics of free and bound ligand molecules, since the structural features of the complex may not be exactly the same in solution and in the solid state [8,9,10,11,12,13,14].

Here, we report on interactions of aminopropyl–azithromycin derivatives **1a**–**c** (Scheme 1), precursors in the synthesis of macrozones, with the ribosome isolated from *E. coli*. Compounds **1a**–**c** also showed promising antibacterial activities against some bacterial strains (Table 1).

We applied NMR techniques such as nuclear Overhauser effect spectroscopy (NOESY) and saturation transfer difference (STD) spectroscopy to study the binding of **1a**–**c** and to determine their ribosome-bound epitopes. Molecular docking was used to characterize complexes of **1a**–**c** with the ribosome and to elucidate the most important sites of interactions.

## 2. Materials and Methods

### 2.1. Antibacterial Testing

Minimum inhibitory concentrations (MICs) were determined according to guidelines of the Clinical Laboratory Standards Institute (CLSI). Compounds were prepared as 10 mg mL^−1^ solutions in dimethyl sulfoxide (DMSO). Final dilutions of the tested compounds were prepared in the 64–0.125 μg mL^−1^ concentration range. The validity of the screen was confirmed by determining MIC values for the reference antibiotic, azithromycin.

### 2.2. NMR

The structure of the prepared precursors was characterized by NMR spectroscopy. One- and two-dimensional NMR spectra were recorded on Bruker Avance III HD 400 and Bruker Avance NEO 600 spectrometers (Bruker Biospin GmbH, Rheinstetten, Germany) equipped with 5 mm inverse (TCI) cryogenically cooled probes and *z*-gradient accessories. Samples were measured at room temperature in CD_3_CN, CDCl_3_ and Tris-d_11_ buffer with TMS as the internal standard. The sample concentration was 20 mg mL^−1^ in CD_3_CN and CDCl_3_ and 2–4 mg mL^−1^ in Tris-d_11_ buffer.

Proton NMR spectra were recorded with 16–128 scans, 11.9 kHz spectral width and a digital resolution of 0.36 Hz per point. For DEPTQ spectra, 3072 scans were used with a spectral width of 35 kHz and digital resolution of 0.67 Hz. In COSY experiments, spectral conditions were: 4–5 scans, 5.6 kHz spectral width, digital resolution of 5.46 Hz per point in f2 and 43.7 Hz per point in f1 dimension. Spectral conditions for edited-HSQC and HMBC spectra were as follows: 9.6 kHz spectral width in f2 and 27.1 kHz in f1 dimension, digital resolution of 4.69 and 212.3 Hz per point in f2 and f1 dimensions, respectively. For both HSQC and HMBC spectra, 4K data points were applied in the time domain and the number of increments for each data set was 256. The number of scans varied between 40 and 64 for HSQC and between 70 and 160 scans for HMBC spectra.

The NOESY spectra were recorded with 50 scans per increment and a relaxation delay of 2 s. Spectral width was 9.6 kHz in both dimensions, and 2 K data points were used in the time domain with 1 K increments in each data set. The digital resolution was 9.39 Hz per point in the f2 dimension and 18.7 Hz per point in the f1 dimension. NOESY spectra were obtained with a mixing time of 400 ms.

In trNOESY experiments recorded in Tris-d_11_ buffer, the same conditions were used except for mixing time (75 ms), number of scans (80), time domain (2 K × 512) and digital resolution in f1 dimension (37.5 Hz per point).

The measuring conditions for ROESY spectra were: 80 scans, 3.2 kHz spectral width in both dimensions, digital resolution of 3.1 and 25.5 Hz per point in the f2 and f1 dimensions, respectively. Data were collected into a 2K × 256 acquisition matrix.

For trNOESY and STD experiments, completely deuterated ribosomes isolated from *E. coli* MRE 600 strain, purchased from Dr. Kalju Vanatalu, Leete Str. 13, 11313 Tallinn, Estonia, were used. The ribosomes were added into the solutions of **1a**–**c** until the final concentration of 1.25 μmol L^−1^ was achieved. STD and trNOESY measurements were carried out with a total volume of 500 μL and a macrolide to ribosome ratio of approximately 3500:1. One-dimensional STD NMR spectra were acquired with 256 scans, 9 kHz spectral width and 3 s saturation time. A total of 32K data points were collected in the time domain. For different precursors, saturation frequency varied, and it was switched from on-resonance (8–11 ppm) to off-resonance (50 ppm) after each scan. Selective saturation of the ribosome was performed using a 50 ms Gauss pulse.

Solid-state NMR spectra were recorded using Bruker Avance Neo 300 and 400 spectrometers equipped with broad-band magic angle spinning (MAS) probes. The samples were spun in 4 mm rotors at the magic angle with 10 kHz. Cross-polarization (CP) MAS NMR experiments were conducted using standard CPMAS pulse sequences with high-power decoupling during acquisition. ^13^C and ^15^N CPMAS NMR spectra were acquired with 16–1024 and 16384 scans, and the recycle delay was 7.0 and 3.5 s, respectively. The spectra were externally referenced to glycine.

### 2.3. Molecular Docking

Using ChemDraw, 3D structures of compounds **1a**–**c** were generated. The ribosome structure was prepared for docking study using the Maestro program, starting from the crystal structure of the *Escherichia coli* ribosome (PDB code: 4V7U). Systems were prepared for docking calculations with Autodock Tools [15] using the default settings and maximum grid size (126 Å × 126 Å × 126 Å). The center of the grid was determined according to the position of the erythromycin molecule in the X-ray structure. Docking calculations were accomplished using Autodock Vina 1.1.2 [16]; exhaustiveness was set to 32. The results were analyzed using Maestro and VMD [17].

## 3. Results and Discussion

The aminopropyl–azithromycin derivatives **1a**–**c** are precursors in the synthesis of macrozones, the novel bioactive azithromycin–thiosemicarbazone conjugates [1,2]. The compounds **1a**–**c** have shown promising activity against some Gram-positive and Gram- negative bacteria, including efflux-resistant *S. pneumoniae* and *S. pyogenes* strains and a Gram-negative *E. coli* strain against which azithromycin is inactive (Table 1).

We employ here a combination of experimental (NMR) and computational methods (molecular docking) to study the structure and interactions of **1a**–**c** with the ribosome isolated from *E. coli*. Prior to binding studies, we identified and elucidated the structures of precursors in solution and solid state.

### 3.1. Structure Elucidation of ***1a**–**c***

To determine the 2D structure of macrozone precursors in solution, we employed one- (^1^H and DEPTQ) and two-dimensional NMR techniques (COSY, edited-HSQC and HMBC). Spectral analysis confirmed the structure as depicted in Scheme 1. It has already been demonstrated that 15-membered macrolides adopt two main conformations in solution, e.g., folded-in and folded-out, referring to the inward or outward folding of the ring fragment C2-C5 (Scheme 1). The percentage of the two conformers also depends on the experimental conditions, such as temperature and solvent polarity [8,9,10].

An intermediate conformation named 3-*endo*-folded-out has also been reported for 15-membered macrolides [10]. Vicinal coupling constants ^3^*J*_H2_,_H3_ and NOE proton–proton contacts H3-H11 and H4-H11 can serve as good indicators of the aglycone ring folding. Hence, the folded-out conformers have larger ^3^*J*_H2,H3_ values (≈10 Hz), larger torsion angles between atoms H2 and H3 (≈ ±180°) and exhibit a close space approach of protons H4 and H11. Lower ^3^*J*_H2,H3_ values (≈2–3 Hz), lower torsion angles (≈100°) and a close contact of atoms H3 and H11 are characteristic of the folded-in conformers [8,11,14].

Several other NOE cross peaks such as H2-H4 and H5-H6Me are indicative of the folded-out structures, and H4-H6Me and H3-H8 are indicative of the folded-in structures. In order to establish whether conformational changes occur upon binding of **1a**–**c** to the ribosome, we first recorded NOESY spectra in a solution of free molecules and measured H2-H3 coupling constants to assess the unbound conformations. We obtained ^3^*J*_H2,H3_ values from the proton spectra in buffered D_2_O and acetonitrile-d_3_ solutions (Table 2) and recorded NOESY spectra to determine distinctive proton–proton NOE connectivities for **1a**–**c** (Table 3). The compound **1b** had a large ^3^*J*_H2,H3_ coupling constant in both solvents (≈10 Hz), suggesting the torsional angle of approximately 180°, and displayed NOE cross peaks characteristic of the folded-out conformation (H4-H11, H2-H4, H5-H6Me). The other two compounds showed smaller ^3^*J*_H2,H3_ values (≈5–6 Hz) intermediate between the two conformational families, suggesting smaller torsional angles, and exhibit H4-H11, but no H3-H11 contacts in buffered D_2_O and acetonitrile-d_3_. This observation is consistent with 3-*endo*-folded-out structures, such as those observed for 15-membered 6-*O*-methyl homoerythromycins [10].

**Figure 1:**
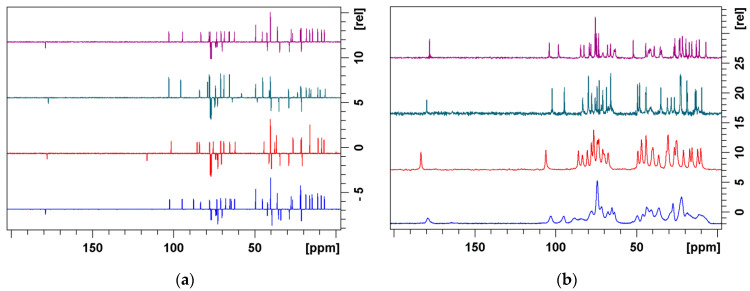
(**a**) Solution−state multiplicity−edited ^13^C DEPTQ and (**b**) solid−state ^13^C CPMAS NMR spectra of azithromycin, **1a**, **1b** and **1c** (from top to bottom).

**Figure 2:**
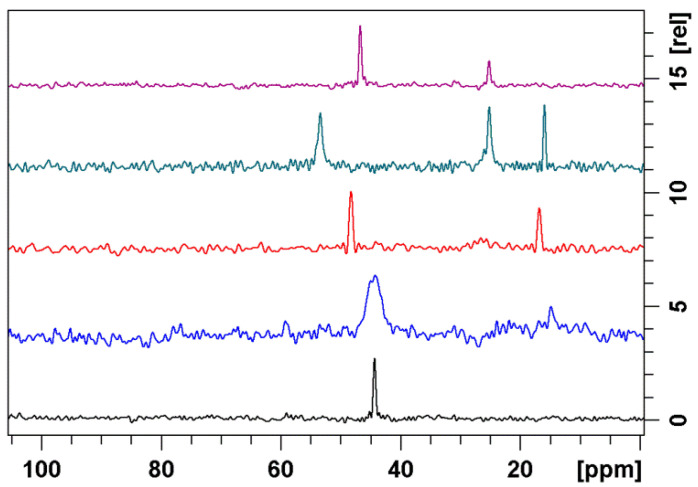
Solid−state ^15^N CPMAS NMR spectra of azithromycin, **1a**, **1b**, **1c** and azithromycin algycone (from top to bottom).

We also recorded ^13^C and ^15^N CPMAS solid-state NMR spectra of the precursors and azithromycin, which are displayed in Figure 1 and Figure 2. CPMAS ^13^C spectra were compared with ^13^C DEPTQ spectra recorded in chloroform-d solution (Figure 1). Substantial line-broadening observed in solid-state spectra clearly demonstrates that 4”-aminopropyl derivative is amorphous, while others are present in ordered crystalline forms. The ^13^C chemical shifts in solid state resemble those obtained in solution, reflecting similar conformations. The analysis of the ^15^N CPMAS spectra also pointed toward the same conclusions.

^15^N chemical shifts of **1a**–**c** can readily be assigned by comparison with those of azithromycin and azithromycin aglycone (Figure 2). Hence, desosamine ^15^N chemical shifts are observed in the region between 45 and 50 ppm, while aminopropyl nitrogen atoms are found the most up-field (around 15 ppm). Nitrogen at position 9a exhibits intermediate chemical shift values (≈25 ppm).

### 3.2. Bound Structures and Interactions with E. coli Ribosome

By using NMR spectroscopy, we sought to establish whether aminopropyl- azithromycin derivatives interact with the *E. coli* ribosome and what are their binding epitopes. We were also interested in possible structural changes that might occur upon the binding to ribosome.

**Figure 3:**
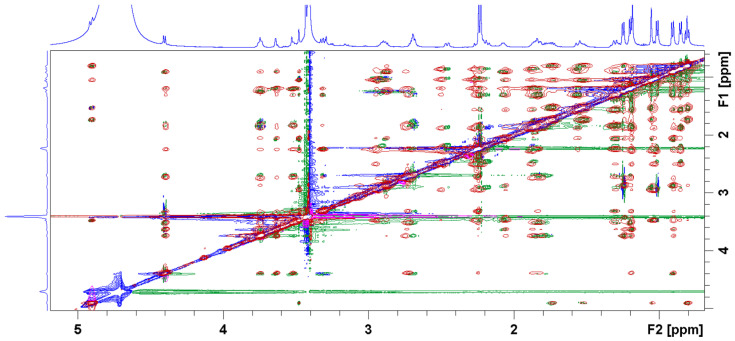
ROESY (blue and green signals) and trNOESY (red and pink signals) spectrum of **1b** in Tris-d_11_ buffer (pH 7.4) prior to and after addition of the *E. coli* ribosome, respectively.

**Figure 4:**
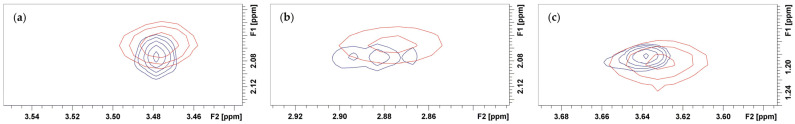
Free (blue) and bound-state (red) NOE cross peaks indicative of the folded-out conformation: (**a**) H4-H11; (**b**) H2-H4; and (**c**) H5-H6Me.

The vast majority of NOE signals observed for **1a**–**c** bound to ribosomes resemble those found in solution indicating similar conformations. The representative ROESY spectrum of **1b** superimposed onto trNOESY spectrum in the presence of ribosome is displayed in Figure 3. It is clearly demonstrated that majority of the cross peaks are identical and that NOE contacts indicative of the folded-out structure such as H4-H11, H2-H4 and H5-H6Me are present in both spectra (Figure 4). TrNOESY spectra of the compounds **1a** and **1c** also resemble NOESY spectra, showing that the dominant bound conformations are 3-*endo*-folded-out. Indicative NOE contacts are displayed in Table 3.

**Figure 5:**
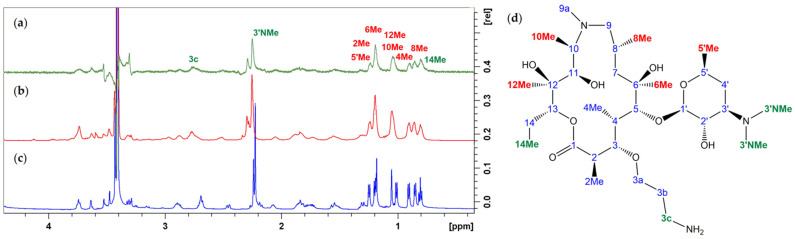
(**a**) STD difference spectrum for **1b** after ribosome addition; (**b**) STD off-resonance spectrum after ribosome addition; (**c**) proton spectrum prior to ribosome addition; (**d**) chemical structure, atom numbering and binding epitopes (green and red) of **1b**.

**Figure 6:**
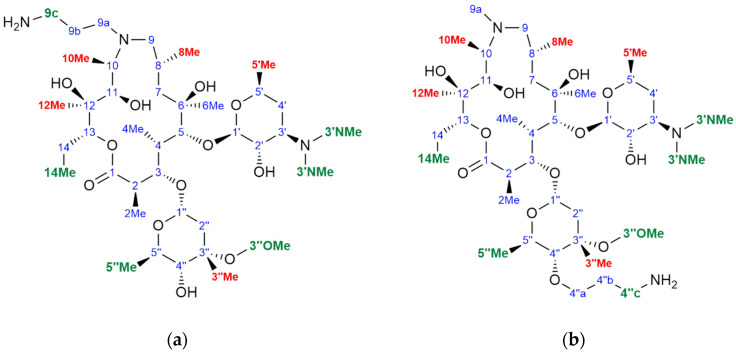
Chemical structure, atom numbering and binding epitopes (green and red) of (**a**) **1a** and (**b**) **1c**.

The compounds **1a**–**c** were further subjected to STD experiments in the presence of *E. coli* ribosomes, to detect structural parts in intimate contact with the ribosome and to determine the binding epitopes. The chemical groups that exhibited STD enhancements are displayed in green and red and are given in Figure 5 and Figure 6. The highest saturation transfers in compound **1b** were observed for methyl groups at positions 3′ and 14 and a methylene group at position 3c (green). An appreciable STD effect was also observed for methyl groups at positions 5′, 6, 8, 10 and 12.

Similarly, the strongest STD enhancements in compounds **1a** and **1c** were observed for the same groups (Figure 6). Saturation transfers were also observed for methyl protons at positions 3″ and 5″ in the cladinose sugar, a moiety that is not present in **1b**.

Hence, trNOESY and STD experiments have shown that all three aminopropyl– azithromycin derivatives bind to the ribosome, and that bound conformations are similar to free ones. A comparison between STD enhancements in **1a**–**c** revealed common regions in close proximity to the ribosomal surface resembling those previously reported for azithromycin and some other 15-membered macrolides [10]. Additional STD effects were observed for methylene protons of the aminopropyl groups, also indicating that NH_2_ protons interact with the ribosome, but they cannot be observed in the spectra owing to exchange with deuterium from the solvent. These new contacts further contribute to the overall binding energy.

### 3.3. Molecular Docking

**Figure 7:**
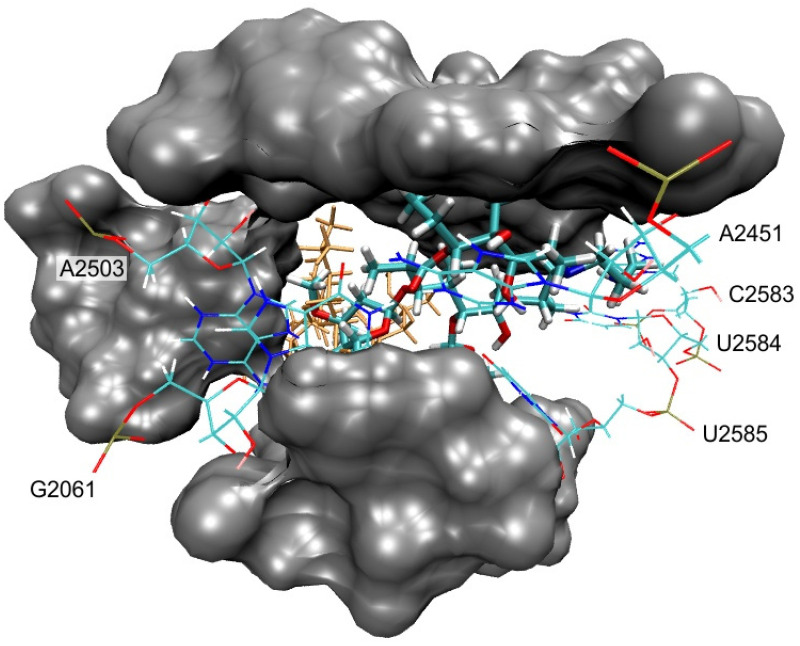
*E. coli* ribosome-binding mode of **1a** that corresponds to the AZI-1 primary binding site [5]. Erythromycin found in the crystal structure (PDB code: 4V7U) is shown as sticks and colored orange. Nucleotides with which compound **1a** forms H-bonds are shown as wires and labeled. Binding site residues forming van der Waals interactions with **1a** (A2058, A2059, A2503, U2504, G2505, U2506, A2062, C2063, C2452, U2584, U2585 and U2586) are shown as grey surfaces.

Docking study revealed that investigated compounds bind to the ribosome in the same binding pocket as erythromycin in the crystal structure of *E. coli* (PDB code: 4V7U). The primary binding sites of **1a** are shown in Figure 7.

**Figure 8:**
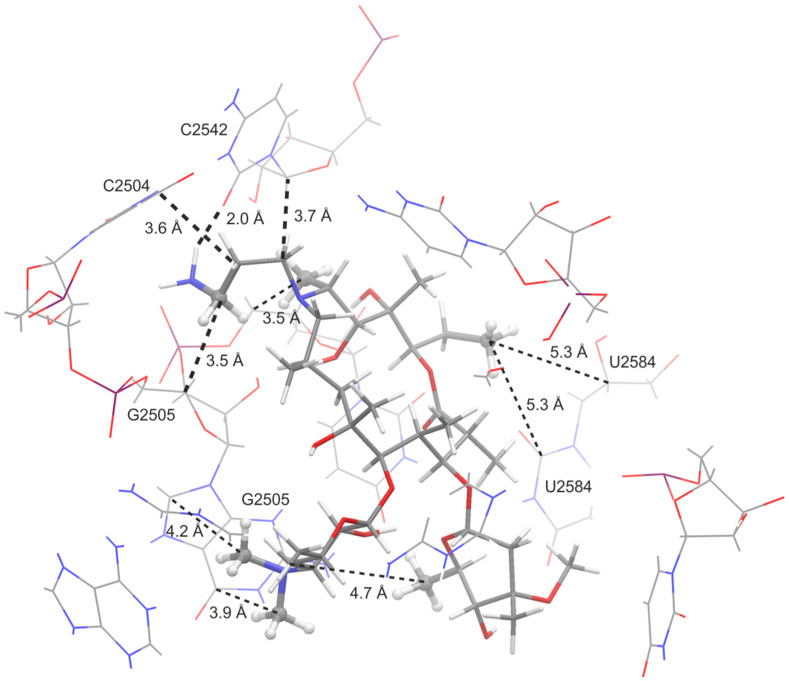
Interactions of **1a** and the main contacts with *E. coli* ribosome nucleotides. The contacts observed in STD experiments and docking calculations are shown as dashed lines, and the interactions between the ribosome and aminopropyl moiety are highlighted as thicker dashed lines.

Also, additional binding sites were detected, but those were less frequent and considered as unspecific binding modes, so we focused on the binding modes that were in the same binding pocket as erythromycin in the crystal structure of *E. coli*. Similar binding modes were found for all three studied azithromycin derivatives and corresponded to the AZI-1 primary binding site as defined by Schlünzen et al. [5]. The macrolactone ring was in a similar position as found in the erythromycin crystal structure (Figure 7), e.g., in the folded-out conformation. Binding was mostly achieved by van der Waals interactions with nucleotides: A2058, A2059, A2503, U2504, G2505, U2506, A2062, C2063, C2452, U2584, U2585 and U2586. The binding specificity of the studied macrozones was achieved through H-bonds with the following nucleotides: A2503, G2061, A2451, C2583, U2584 and U2585 (Figure 7). In some binding modes, aminopropyl moiety enhances binding by making additional interactions with the ribosome; these contacts are highlighted in Figure 8. Although similar interactions with the ribosome were computationally found for all three compounds, their binding affinities differed. More favorable binding affinities in the erythromycin binding pocket were calculated for compounds **1a** (−10.2 kcal/mol) and **1c** (−9.6 kcal/mol) than for compound **1b** (−8.3 kcal/mol). Differences in calculated binding affinities were in accordance with the experimental differences in activities against *E. coli* (Table 1).

## 4. Conclusions

We have demonstrated that NMR spectroscopy and docking study can provide valuable data on the conformation and binding interactions of aminopropyl– azithromycin derivatives, precursors in the synthesis of bioactive macrozones. All compounds adopted similar conformations in free and bound states. Binding epitopes resemble those found for erythromycin, azithromycin and related 15-membered macrolides. These results can further be exploited in the process of designing novel macrolide derivatives with activity against resistant pathogens.

## Figures and Tables

**Table 1:** In vitro antibacterial activity (MIC, μg/mL) for aminopropyl–azithromycin derivatives **1a**–**c**.

Compound	*S. pneumoniae* B0652	*S. pneumoniae* B0326	*S. pyogenes* B0542	*S. pyogenes* B0545	*S. aureus* ATCC 29213	*E. faecalis* ATCC 29212	*M. catarrhalis* ATCC 23246	*E. coli* ATCC 25922
	eryS	M	eryS	M	eryS			
	MIC (μg/mL) ^a^							
**1a**	1	64	0.5	64	8	64	4	32
**1b**	16	64	32	64	>64	>64	2	64
**1c**	0.5	16	2	32	16	64	2	32
**azithromycin**	≤0.125	16	≤0.125	8	1	8	≤0.125	>64

^a^ minimum inhibitory concentration.

**Table 2:** Vicinal coupling constants ^3^*J*_H2_,_H3_ for **1a**–**c** in free state.

Compound	^3^*J*_H2_,_H3_/Hz	
	Acetonitrile	Tris Buffer
**1a**	6.60	6.43
**1b**	10.14	9.88
**1c**	5.39	5.11

**Table 3:** Characteristic NOE contacts for **1a**–**c** in free and bound states.

NOE Contact	Compound								
		1a			1b			1c	
	acet. ^a^	Tris ^b^	rib. ^c^	acet. ^a^	Tris ^b^	rib. ^c^	acet. ^a^	Tris ^b^	rib. ^c^
H3-H11	−	−	−	−	−	−	−	−	−
H4-H11	+	+	+	+	+	+	+	+	+
H2-H4	+	+	+	+	+	+	+	+	+
H4-H6Me	−	−	−	−	−	−	−	−	−
H3-H8	−	−	−	−	−	−	−	−	−
H5-H6Me	+	+	+	+	+	+	+	+	+
H8-H11	−	−	−	−	−	−	−	−	−

^a^ free state in acetonitrile-d_3_ solution; ^b^ free state in Tris-d_11_ buffer solution; ^c^ ribosome-bound state.
